# Does the interval from melphalan administration to infusion of hematopoietic stem cells affect outcomes after autologous transplantation in multiple myeloma?

**DOI:** 10.1016/j.htct.2026.106533

**Published:** 2026-09-26

**Authors:** Viktoria Weihermann, Paulo Victor Rabelo Barbosa, Maria Eduarda Alonso Joaquim de Carvalho, Sabrina Maria Corrêa Brant Pereira de Jesus, José Vivaldo Moreira Feitosa Júnior, Vinicius Burnett Aboud Souza da Eira, Martha Mariana de Almeida Santos Arruda, Volney Assis Lara Vilela, André Domingues Pereira

**Affiliations:** Instituto de Cardiologia e Transplantes do Distrito Federal, Brasília, Brazil

To the editor:

Multiple myeloma (MM) is a hematologic malignancy with significant morbidity and mortality. Autologous hematopoietic stem cell transplantation (ASCT) remains a key therapy, improving progression-free survival [1]. Conditioning is typically performed with high-dose melphalan (200 mg/m²) [2]. Despite its short half-life (∼60 min) and pharmacokinetic data suggesting stem cell infusion should occur within eight hours, most protocols use intervals >24 h due to concerns about cytotoxic effects on progenitor cells and potential impairment of engraftment [3]. Consequently, the optimal timing between melphalan administration and stem cell infusion remains uncertain, with prior studies yielding inconsistent results [4, 5, 6, 7]. This question is particularly relevant in low- and middle-income settings, such as Brazil, where timing may influence hospitalization duration and costs.

In this retrospective, single-center study, patients who underwent ASCT between January 2023 and March 2025 were screened for inclusion. The primary objective was to assess whether stem cell infusion <24 h after melphalan administration affected time to neutrophil engraftment. Secondary objectives included platelet engraftment, length of hospitalization, and treatment-related toxicities. Neutrophil engraftment and platelet engraftment were established according to standard criteria [8]. All adult patients with MM or other plasma cell disorders who received single-agent melphalan as conditioning were included in this analysis. Patients underwent stem cell mobilization with granulocyte colony-stimulating factor (G-CSF), with or without plerixafor. Peripheral blood stem cells were collected from all patients and infused as fresh, non-cryopreserved products. For patients with chronic kidney disease with clearance of creatinine <30 mL/min, age above 70 years and frail patients, the dose of melphalan was reduced to 140 mg/m^2^. Dose was also adjusted for obese patients (defined as >120% above ideal body weight), in which adjusted body surface was used. G-CSF was administered in the post-transplantation period beginning on Day +5 and continued until neutrophil recovery.

The patients were divided into two groups based on the timing of stem cell infusion after melphalan administration (<24 h in Group 1 versus ≥24 h in group 2). The observed differences in the time intervals were a consequence of the institutional protocol, which schedules stem cell infusion exclusively during the morning for logistical reasons. The study was approved by the local ethics committee (CAAE 88,565,925.6.0000.0026).

A total of 115 patients were evaluated: 59 received the stem cell infusion <24 h after melphalan administration, and 56 received it ≥24 h after administration. Baseline characteristics are summarized in Table 1 and were well balanced between groups. Disease status at transplantation and CD34^+^ cell dose did not differ significantly between cohorts. The median interval between the end of melphalan infusion and the start of stem cell infusion was 19 (18–23) hours in Group 1 and 24 (24–50) hours in Group 2.

**Table 1:** Patient characteristics.

Variable	Group 1 (<24 h)	Group 2 (≥24 h)	p-value
Median age (years) - median (range)	58 (42–73)	61.5 (33–76)	0.370,634
Sex - % Female Male	44% 56%	41% 59%	0.774,913
Disease status – n (%) PR VGPR CR	14 (23.7%) 41 (69.5%) 4 (6.8%)	12 (21.4%) 35 (62.5%) 9 (16.1%)	0.945,585
CD34 stem cell dose infused - median × 10^6^/kg	4.47	4.32	0.6765
Conditioning - % Melphalan 200 mg/m^2^ Melphalan 140 mg/m^2^	83% 17%	81% 19%	0.854,203
Median time between melphalan and infusion (hours) – median (range)	19 (18–23)	24 (24–50)	<0.001

PR: Partial Response; VGPR: Very Good Partial Response; CR: Complete Response.

None of the characteristics differed significantly between the two study groups (except for the median time between melphalan administration and infusion).

The Mann-Whitney test was used for continuous measures and Fisher’s exact test and Qui-square test were performed for categorical measures. All reported p-values are 2-sided, with p < 0.05 considered significant. Statistical analyses were performed using GraphPad Prism® (version 10.6.1).

Neutrophil and platelet engraftment were achieved in all but two patients who died on Days +9 and +10 post-ASCT due to septic shock. Median times to neutrophil and platelet engraftment were similar between the groups. These findings remained unchanged when analyzing only patients who received full dose melphalan (200 mg/m^2^). Length of hospitalization did not differ significantly between groups (Table 2); incidence of febrile neutropenia was also similar (p = 0.98). Notably, no hospital readmissions were observed following discharge.

**Table 2:** Transplant outcomes.

	Group 1 (<24 h)	Group 2 (≥24 h)	p-value
Days to neutrophil engraftment – median (range)	11 (9–15)	11 (9–16)	0.09,492
Days to platelet engraftment - median (range)	11 (8–20)	11 (8–20)	0.17,384
Days of hospitalization - median (range)	14 (12–48)	15 (12–49)	0.213

The nonparametric Mann Whitney test was used to compare engraftment and length of hospitalization. All reported p-values are 2-sided, with p < 0.05 considered significant. Statistical analyses were performed using GraphPad Prism® (version 10.6.1).

Another key outcome assessed was the incidence of mucositis. Given missing data and inconsistencies between the groups, these results should be interpreted with caution. The incidence of mucositis was 65% in Group 1 and 74% in Group 2; however, a higher proportion of patients in Group 1 had missing data (20% vs 12.5% in group 2; p = 0.308). No differences in mucositis severity were observed, with only one case of grade 4 reported in each group.

In summary, engraftment kinetics and length of hospitalization did not differ between the groups in this study. Although prior reports have associated shorter dosing intervals with prolonged hospitalization or increased infection-related readmissions, this pattern was not observed in the present cohort. The main limitations of this study include the retrospective design, small sample size, and single-center setting, which might increase the possibility of type II errors. Moreover, the actual difference between groups was relatively small (median 19 vs. 24 h), which may reduce the ability to detect clinically meaningful differences. Larger, prospective, multicenter studies are warranted to better define the optimal dosing schedule.

## Funding

The authors declare that this study did not receive any specific grant from funding agencies in the public, commercial, or not-for-profit sectors.

## Ethics approval

This study was approved by the local ethics committee (CAAE: 88,565,925.6.0000.0026)

## Data availability statement

The data that support the findings of this study are available from the corresponding author upon reasonable request.

## Conflicts of interest

The authors declare no conflicts of interest.
